# Incidence and Viral Etiology of Acute Respiratory Infections and Pneumonia among Children Under Two Years: A Birth Cohort Study in Dhaka, Bangladesh

**DOI:** 10.4269/ajtmh.25-0466

**Published:** 2025-12-04

**Authors:** Ashrak Shad Pyash, Homayra Rahman Shoshi, Md. Abdullah Al Jubayer Biswas, Mustafizur Rahman, Rashidul Haque, William A. Petri, Fahmida Chowdhury, Md. Zakiul Hassan

**Affiliations:** ^1^ Infectious Diseases Division, International Centre for Diarrhoeal Disease Research, Bangladesh (icddr,b), Mohakhali, Dhaka, Bangladesh;; ^2^ Collaborative Biostatistics Program, University of Saskatchewan, Saskatoon, Canada;; ^3^ Division of Infectious Diseases and International Health, University of Virginia, Charlottesville, Virginia;; ^4^ Pandemic Sciences Institute, Nuffield Department of Clinical Medicine, University of Oxford, Oxford, United Kingdom

## Abstract

Globally, acute respiratory infections (ARI), including pneumonia, remain the leading infectious causes of morbidity and mortality among children under-two years of age. We conducted this longitudinal birth cohort study in a low-income urban community in Dhaka, Bangladesh, to estimate the incidence of ARI and pneumonia and assess their viral etiology. From May 2015 to March 2016, 447 children were enrolled and followed till 2022. In this analysis, we included data from the first two years of children’s lives, which contributed to a total observation of 778 child-years. Nasopharyngeal wash samples were collected during symptomatic episodes, which were tested using rRT-PCR for rhinovirus (RV), respiratory syncytial virus (RSV), human metapneumovirus (hMPV), influenza virus, human parainfluenza virus (HPIV), and adenovirus. We calculated incidence rates using Poisson-based methods with 95% confidence intervals (CI) and stratified age-specific rates into three groups: 0 to <6 months, 6 to <12 months, and 12 to 24 months. A total of 2,335 ARIs and 314 pneumonia episodes were documented. At least one respiratory virus was detected in 71% of ARI and 75% of pneumonia episodes. RV was the most frequently detected virus (54% in ARI, 40% in pneumonia), followed by RSV, HPIV, and influenza. The incidence of viral ARI was 212/100 child-years (95% CI: 202–223), and that of viral pneumonia was 30/100 child-years (95% CI: 27–35). The observed incidence of viral ARI and pneumonia during early childhood underscores the need for targeted interventions. Future research should examine environmental and socioeconomic influences, assess preventive strategies, and improve early detection and treatment.

## INTRODUCTION

Acute respiratory infections (ARI), including pneumonia as a severe manifestation, represent a significant cause of morbidity and mortality among children under 5 years of age.^1^^,^^2^ Children across socioeconomic backgrounds experience four to five episodes of ARI annually. These episodes account for nearly half of all global pediatric health care visits, including both hospital admissions and outpatient consultations.^3^^–^^5^ According to the WHO, ARIs are responsible for at least 15% of mortality among children under 5 years of age globally,^6^ with pneumonia alone accounting for 14% of all such deaths.^7^ In 2012, ARI caused approximately 3.9 million child deaths globally.^5^^,^^8^ On the other hand, pneumonia is responsible for nearly 0.7 million deaths among children under 5 years old, 68% of which occur in infants.^7^

The burden of ARIs and pneumonia is disproportionately higher in low- and middle-income countries (LMICs) in comparison with high-income countries. In Africa and Asia, ARI accounts for approximately 70% of total deaths in children under 5 years of age.^1^^,^^9^^,^^10^ Four South Asian countries—Bangladesh, India, Indonesia, and Nepal—collectively contribute to 40% of global childhood ARI mortality.^11^ Additionally, South Asia also reports the highest pneumonia incidence rate, with 2,500 cases per 100,000 children.^12^

In Bangladesh, ARIs and pneumonia remain leading health burdens for children under 5 years of age.^13^ Moreover, ARI accounts for one in four deaths in children under 5 years old in the country,^14^ whereas nearly 87% of Bangladeshi children experience at least one ARI episode during their first year of life.^15^ On the other hand, more than 10 million new pneumonia cases are diagnosed each year among children of this age group in Bangladesh.^16^ In 2015, pneumonia alone was responsible for more than 12,000 child deaths annually in the country, contributing to ∼15% of total mortality in children under 5 years old.^17^

Although bacterial pathogens have traditionally been the primary focus of pediatric ARI and pneumonia treatment, growing evidence highlights the significant contribution of viral pathogens to the overall disease burden.^18^^–^^21^ Primary respiratory viral infections can increase children’s susceptibility to secondary bacterial infections, most commonly with *Streptococcus pneumoniae* and *Haemophilus influenzae*.^10^ Advances in molecular diagnostics have identified viruses such as respiratory syncytial virus (RSV), influenza viruses (A and B), human parainfluenza viruses (HPIV), rhinovirus, adenovirus, and human metapneumovirus (hMPV)^10^^,^^22^^,^^23^ as key contributors to ARI and pneumonia in young children.

Although global and national estimates often focus on children under-five, infants, particularly those under 2 years of age, remain underrepresented in epidemiological data. Moreover, data specific to viral ARI and pneumonia among children under 2 years also remain limited. This gap is especially concerning in an LMIC like Bangladesh, where limited access to viral diagnostics hinders accurate assessment of infections. To address this gap, we conducted this longitudinal study to estimate the community-based incidence of ARI and pneumonia from laboratory-confirmed respiratory viruses in a low-income urban setting of Dhaka, Bangladesh. By generating age-specific viral ARI and pneumonia burden data, this study can guide targeted interventions and support progress toward Sustainable Development Goal (SDG) 3.2.^24^

## MATERIALS AND METHODS

### Study site.

**Figure 1.**
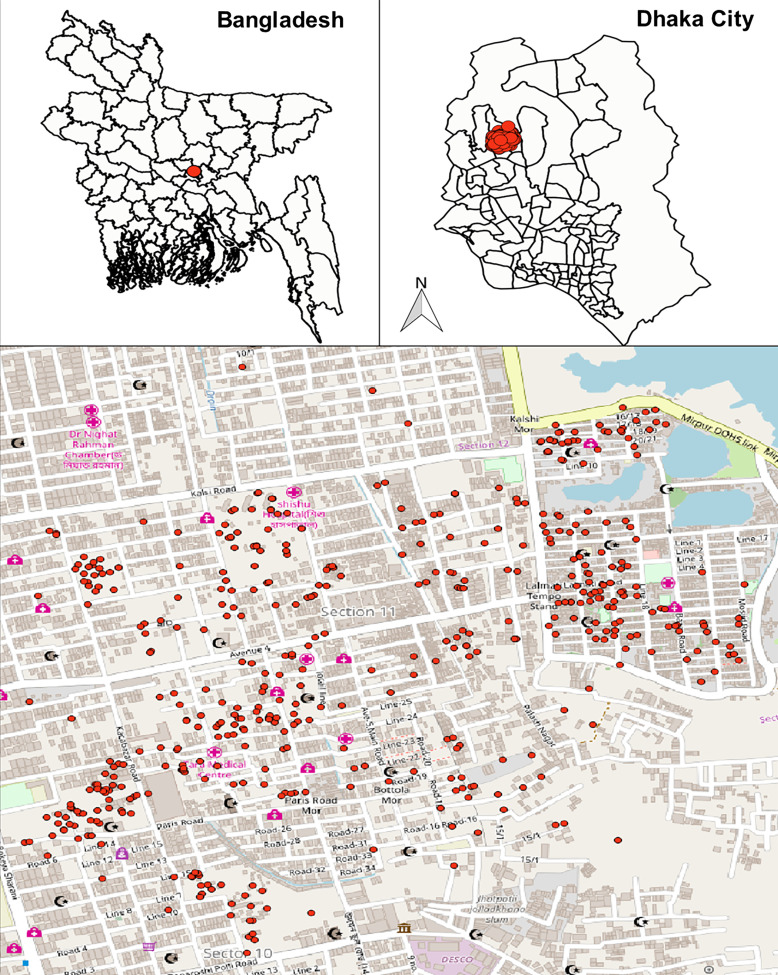
Household distribution of enrolled participants.

Our study was conducted in a densely populated, low-income, urban slum within Mirpur Thana (commonly known as the “Mirpur Slum”) located in the northwestern region of Dhaka, Bangladesh. Mirpur Thana (“Thana” is the smallest administrative unit in Bangladesh) is one of Dhaka’s 50 thanas and spans approximately 59 km^2^. The area is subdivided into 14 sections, with our study focused specifically on Section 11 (Figure 1). At the time of the study, the population of Mirpur Section 11 was estimated to be ∼50,000, with minimal migration. The slum is characterized by narrow alleys, overcrowded living conditions, and limited access to basic municipal services. The community mainly consists of low-income residents engaged in informal or daily wage labor, residing in predominantly informal housing structures.

### Study settings.

In May 2015, the International Centre for Diarrheal Disease Research, Bangladesh (icddr,b) initiated this respiratory infection cohort study to assess respiratory virus–associated infections among young children in the study area. This study leveraged an existing enteropathogen birth cohort, which had begun enrolling infants during their first week of life in June 2014. At the onset of this respiratory infection cohort, families previously enrolled in the enteropathogen study were approached for re-consent and subsequent enrollment into this study.

**Figure 2.**
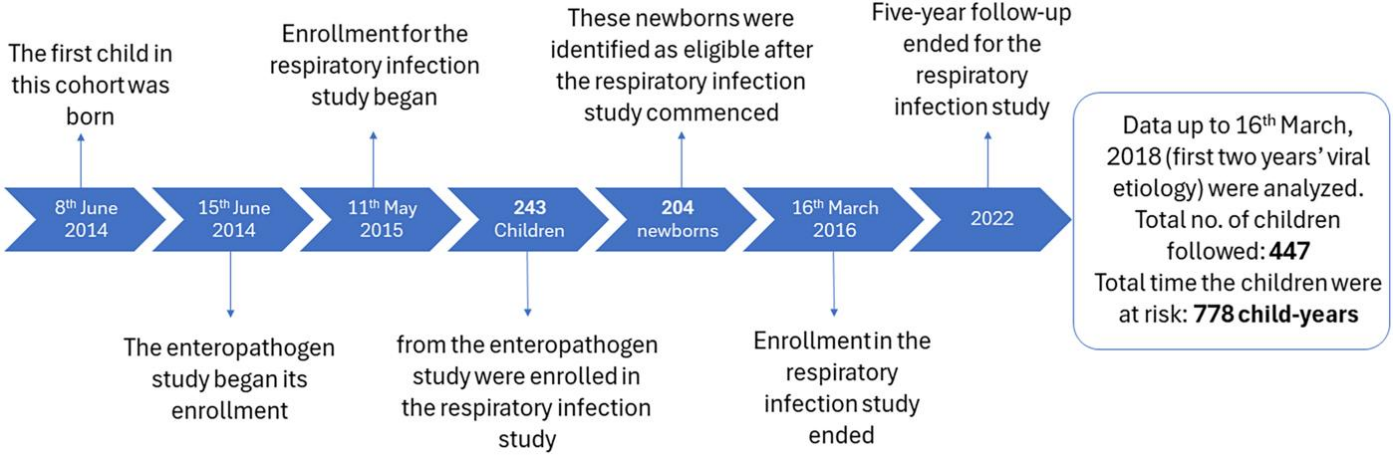
Timeline of participant recruitment.

The enteropathogen study field team identified pregnant women in their third trimester and newborns within their first week of life. All children born within a 1.5-km radius of the study area were eligible for enrollment into the study. Following the initial enrollment into the respiratory infection cohort in May 2015, the population was treated as an open cohort, with continuous enrollment of all eligible children born in the study area until March 2016 (Figure 2). All enrolled children were followed for 5 years until 2022. However, in this paper, we report only the burden of viral respiratory illnesses for the first 2 years of life. Field staff conducted household visits twice weekly (approximately every 3 days) to identify symptoms of respiratory infections. Additionally, parents were advised to bring their child to the study clinic set up in the area whenever they observed signs of ARIs.

### Inclusion and exclusion criteria in the study.

Enrollment of the children into this cohort followed a clearly defined eligibility criterion. Infants were included only if their parents provided written informed consent, the child was between 0 and 7 days of age, had no visible congenital deformities, and the household was considered stable with no plans to relocate within the following year. Exclusion criteria included parental refusal to allow nasal wash sample collection from the child, plans to enroll the child in another interventional clinical study during our study period, unwillingness to permit twice-weekly home visits by the field staff, a history of seizures or any other apparent neurological disorders in the infant, or if the infant had a sibling or a twin enrolled in the same study.

### Data collection.

At baseline, trained study staff collected the newborn’s anthropometrics (age, height, weight) and demographic information, including the household’s socioeconomic status (parental education level, family income, etc.) during enrollment.

During the follow-up period, field staff conducted household visits twice weekly to monitor the health status of each enrolled child. At each visit, caregivers were interviewed using a standardized symptom checklist to ascertain whether the child had experienced any respiratory symptoms within the past 72 hours. Symptoms included sudden or subjective fever; cough; sore throat; difficulty breathing; rapid, labored, or noisy breathing; chest indrawing when calm; nasal discharge; ear pain; reluctance or inability to drink; convulsions; lethargy; decreased activity; and repeated vomiting.^25^ When children with any respiratory symptoms were identified during a household visit by the study team, they were referred to the study clinic for further clinical evaluation. Nasopharyngeal wash samples were collected only from children presenting with respiratory symptoms identified during scheduled household follow-up visits. No routine samples were collected from asymptomatic children.

At the study clinic, physicians used a structured questionnaire to record a detailed history of the illness episode, including symptom onset, duration, and severity. A clinical examination was performed for every child presenting with a new episode of subjective or measured fever (a temperature ≥38°C) or any respiratory symptom such as cough, nasal discharge, or breathing difficulty. A new episode of ARI or pneumonia was defined as one preceded by at least 7 consecutive symptom-free days.

For each new episode, study physicians conducted a standardized physical examination. Nasopharyngeal wash samples were collected for laboratory testing to identify viral pathogens. Nasopharyngeal wash samples were used to collect respiratory samples from the children because they provide a higher yield of respiratory viruses than swabs, especially in young children with small nasal passages. The method is less dependent on precise technique, can collect a larger volume of secretions, and enhances diagnostic sensitivity for respiratory pathogens.^26^ Clinical diagnoses were made following WHO guidelines: children presenting with cough or nasal discharge were diagnosed with acute respiratory infection (ARI). On the other hand, pneumonia was diagnosed in children who had cough or breathing difficulty along with age-specific tachypnea, defined as a respiratory rate ≥60 breaths per minute for infants ages 0 to <2 months, ≥50 for infants age 2 to <12 months, and ≥40 for children age 12 to 24 months.^27^ The study clinic provided all clinical care, diagnostic services, and referrals for higher-level medical management, including hospitalization when required, free of cost. Children needing tertiary-level care were referred to designated public or private hospitals where the study team maintained referral links.

### Sample collection and testing.

Using a sterile technique, trained study physicians collected nasopharyngeal wash specimens from children. The procedure involved using a 10-mL syringe prefilled with 5 mL of sterile normal saline, which was introduced into the nasopharynx via a butterfly catheter. Children were positioned in a 30° semi-Fowler position with the head slightly inclined forward to optimize sample retrieval. The catheter was inserted approximately 2–3 cm into the nasal passage, saline was instilled, and immediate suction was applied as the catheter was withdrawn.^25^

Collected specimens were promptly transferred into viral transport medium containing Dulbecco’s Modified Eagle Medium (DMEM) and stored at 4°C at the field site. Samples were transported daily to the virology laboratory at the icddr,b within 8 hours of collection and stored at −70°C until further processing. Respiratory viral pathogens, including influenza viruses, RSV, adenovirus, HPIV, RV, and hMPV, were detected using real-time reverse transcription polymerase chain reaction (rRT-PCR) assays with primers and probes supplied by the U.S. Centers for Disease Control and Prevention (CDC), Atlanta, Georgia.^28^ However, detection of RV was incorporated into the respiratory virus panel in May 2016.

## STATISTICAL ANALYSES

All data were collected using electronic tablets and transferred to STATA 15 (StataCorp, College Station, TX) for data cleaning and statistical analysis. The length of time between each child’s date of enrollment and their last visit (either at the 2-year follow-up or prior to loss to follow-up) was used to determine the child-years at risk. All statistical analyses, including incidence rate estimation and confidence intervals, were conducted using Poisson-based methods.

Incidence rates were calculated by dividing the number of new episodes of ARI or pneumonia by the total child-time at risk, expressed in child-years. Children were eligible to contribute multiple episodes, provided that a minimum of 7 symptom-free days separated each event to ensure independence. Person-time was calculated from the date of enrollment to the date of censoring. For virus-specific incidence rate estimation, only episodes with confirmed viral detection in the nasopharyngeal wash samples were included in the numerator. Corresponding person-time was included in the denominator only during periods when the specific virus was actively tested. Because RV testing was introduced in May 2016, RV incidence was calculated using only follow-up time from that point, excluding any earlier period during which RV was not tested.

Incidence rates were stratified by age group (0 to <6 months, 6 to <12 months, 12 to 24 months), based on the child’s age at the time of illness onset. All rates are presented per 100 child-years of follow-up, with exact 95% confidence intervals calculated using Poisson methods. Episodes in which two or more respiratory viruses were detected in the same nasopharyngeal sample were classified as “multiple viruses” and counted as a single episode. This grouping approach was used to avoid double-counting the same episode under multiple individual virus categories and to ensure that each ARI or pneumonia episode contributed only once to the overall incidence analysis.

### Ethical approval.

Before enrollment in the respiratory infection study, we obtained written informed consent from each child’s parents or adult primary caregiver. The study protocol was reviewed and approved by the institutional review board at the icddr,b. The institutional review board at US CDC deferred to the icddr,b approval.

## RESULTS

### Sociodemographic and baseline characteristics.

We enrolled a total of 447 children in our study between May 2015 and March 2016 and followed them up to 2 years of their age. Of the enrolled children, 243 were born prior to the initiation of the study, whereas 204 were born after the study had commenced. The total time the 447 children were under observation in this study was 778 child-years. Additionally, our participants had a median age of 6 days at enrollment (interquartile range [IQR]: 4–116 days) into this study. The median birth weight of the children was 2,740 g (IQR: 2,490–3,020 g). More than half (54%) of the children in our study were female and came from nuclear families (56.2%). Seven out of 10 parents were educated (70%). The reported median monthly family income and household members were 13,000 Taka and 5, respectively (Table 1). Approximately 67% of the infants’ families resided in semipermanent housing structures, and 48% of the children lived in households where the kitchen was located indoors.

**Table 1:** Sociodemographic and baseline characteristics of the study participants in the cohort, Dhaka, Bangladesh (May 2015–February 2018)

Characteristics	*N* = 447 *n* (%)
Median age in days at enrollment (IQR)	6 (4–116)
Sex	
Male	206 (46.1)
Female	241 (53.9)
Nutritional status	
Children with height for age *z*-score (HAZ) ≤2	
Yes	86 (19.2)
No	361 (80.8)
Children with weight for age *z*-score (WAZ) ≤2	
Yes	63 (14.1)
No	384 (85.9)
Feeding practice at birth	
Breast milk	394 (88.1)
Cow or goat milk	18 (4.03)
Breast milk and cow or goat milk	6 (1.34)
Water or sugar or honey	29 (6.49)
Parental education	
Father without formal education	
Yes	137 (30.7)
No	310 (69.3)
Mother Without Formal Education	
Yes	97 (21.7)
No	350 (78.3)
Family characteristics	
Family type	
Nuclear	251 (56.2)
Joint	196 (43.8)
Family income (Taka)	
Median (IQR)	13,000 (9,500–18,000)
≤13,000	229 (51.2)
>13,000	218 (48.8)
Number of members in the family	
Median (IQR)	5 (4–6)
≤4	185 (52.9)
≥5	165 (47.1)
Environmental exposure	
Exposed to tobacco smoke	
Yes	258 (57.7)
No	189 (42.3)
Type of house*	
Semipermanent	294 (65.8)
Permanent	153 (34.2)
Use of clean fuel^†^	
Yes	149 (33.3)
No	298 (66.7)
Location of kitchen	
Inside house	214 (47.8)
Separate building	8 (1.79)
Outdoors	225 (50.3)

IQR = interquartile range.

* Semipermanent house = roof: tin/straw/rudimentary, floor: bamboo-wood/earth; permanent house = roof: concrete, floor: cement tiles.

^†^ Clean fuel = electric and gas stove.

### Clinical manifestation and laboratory-confirmed respiratory viral pathogen.

Of the 447 children enrolled in the study, 442 (99%) experienced at least one episode of respiratory illness within their first 2 years of age, resulting in a total of 2,682 respiratory infection episodes during the follow-up period. Among these, 2,335 (87%) episodes were clinically diagnosed as ARI, and 314 (12%) were classified as pneumonia by the study physicians based on clinical assessment. The remaining 33 episodes (1%) were diagnosed as “only cough” by the physicians.

In this cohort, 424 (95%) children experienced at least one episode of ARI during the study period. Of these, 50 (12%) children had one episode, 59 (14%) had two episodes, 62 (15%) had three, 52 (13%) had four, and the remaining 201 (47%) experienced five or more episodes. The median age at onset of the first ARI episode was 4 months (IQR: 2–5 months). Notably, 416 (98%) children received antibiotics at the time of diagnosis in the study clinic.

Additionally, 163 children experienced at least one episode of pneumonia. Among these, 49 children (30%) had two pneumonia episodes, 20 (12%) had three, and seven children (4%) experienced four episodes. The median age of children at the time of developing the first pneumonia episode was 4.6 months (IQR: 2.2 7.4 months), and 162 (99%) were treated with antibiotics at diagnosis.

At least one respiratory virus was detected in 71% (1,652 out of 2,335) of all the ARI episodes. Similarly, viral pathogens were identified in 75% (236 out of 314) of the pneumonia episodes. RV was the most common virus in the nasopharyngeal wash samples obtained in the study, accounting for 54% and 40% of episodes of all-aged ARI and pneumonia, respectively. RSV was the second most frequent virus, detected in 10% of samples obtained from children with ARI and 17% of those with pneumonia. HPIV was identified in 8% of ARI and 17% of pneumonia episodes, followed by influenza in 7% ARI and 4% of pneumonia episodes. Two or more respiratory viruses were detected in approximately 13% of the ARI and pneumonia cases (Table 2). In cases of multiple viruses detected, co-detection of RV and RSV was the most frequently identified viral combination in both ARI and pneumonia episodes (Tables S1 and S2).

**Table 2:** Viral etiology of ARI and pneumonia in children under 2 years of age in a low-income urban community, Dhaka, Bangladesh (May 2015–February 2018)

Viral Pathogens	Viral ARI Episodes (*N* = 1652)				Viral Pneumonia Episodes (*N* = 236)			
	All Ages *n* (%)	0 to <6 Months *n* (%)	6 to <12 Months *n* (%)	12 to 24 Months *n* (%)	All Ages *n* (%)	0 to <6 Months *n* (%)	6 to <12 Months *n* (%)	12 to 24 Months *n* (%)
Rhinoviruses (RV)	884 (53.5)	179 (53.1)	225 (51.8)	480 (54.5)	94 (39.8)	27 (35.5)	28 (36.8)	39 (46.4)
Respiratory syncytial virus (RSV)	171 (10.4)	24 (7.12)	49 (11.3)	98 (11.1)	41 (17.4)	15 (19.7)	15 (19.7)	11 (13.1)
Human parainfluenza viruses (HPIV)	135 (8.2)	34 (10.1)	48 (11.1)	53 (6.02)	41 (17.4)	13 (17.1)	20 (26.3)	8 (9.52)
Influenza viruses	113 (6.8)	26 (7.72)	31 (7.14)	56 (6.36)	9 (3.81)	4 (5.26)	1 (1.32)	4 (4.76)
Adenoviruses	71 (4.3)	7 (2.08)	26 (5.99)	38 (4.31)	6 (2.54)	1 (1.32)	3 (3.95)	2 (2.38)
Human metapneumovirus (hMPV)	61 (3.63)	17 (5.04)	3 (0.69)	41 (4.65)	15 (6.36)	7 (9.21)	1 (1.32)	7 (8.33)
Multiple viruses	217 (13.1)	50 (14.8)	52 (11.9)	115 (13.1)	30 (12.7)	9 (11.8)	8 (10.5)	13 (15.5)
Total No. of episodes	1,652 (100%)	337 (100%)	434 (100%)	881 (100%)	236 (100%)	76 (100%)	76 (100%)	84 (100%)

Among children diagnosed with pneumonia, the most commonly reported symptoms (aside from cough and runny nose in 100% of them) were fever (68.2%), noisy breathing (40.4%), and difficulty breathing (34.4%). Additional symptoms included chest indrawing when calm (23.9%), whistling in the chest (15.3%), decreased activities (11.8%), and repeated vomiting (2.4%), indicating the presence of more severe respiratory distress in these episodes. The frequency and distribution of common symptoms did not vary between ARI and pneumonia episodes caused by different viral pathogens (Supplemental Figures 1 and 2).

### Incidence of virus-associated ARI and pneumonia.

The incidence of ARI in our cohort of children was 302/100 child-years (95% CI: 290–314), and that of pneumonia was found to be 40/100 child-years (95% CI: 36–45). Children age 0 to <6 months were followed for 112 child-years at risk, 6 to <12 months for 253 child-years at risk, and 12 to 24 months for 413 child-years at risk. We found that the overall yearly incidence of ARI and pneumonia in our cohort of children related to laboratory-confirmed respiratory viral pathogens were 212 episodes/100 child-years and 30 episodes/100 child-years, respectively.

The overall annual incidence of viral ARI was higher in children age <6 months (344 episodes/100 child-years) in comparison with children age 6 to <12 months (229 episodes/100 child-years) and children age 12 to 24 months (180 episodes/100 child-years) (Table 3). A similar trend was also observed in the case of pneumonia, and children age <6 months (76 episodes/100 child-years) had a greater incidence than the other two groups. Furthermore, we also assessed the yearly incidence of specific respiratory viruses detected in ARI and pneumonia among our cohort. For ARI, the annual incidence among children <6 months of age ranged from 137 episodes/100 child-years for RV to 9 episodes/100 child-years for adenovirus. For pneumonia, it was 12 episodes/100 child-years for RV to 1 episode/100 child-years for adenovirus. The highest incidence of RV-associated ARI and pneumonia was observed among children age <6-months, which had a reduced person-time denominator due to the later introduction of RV in our respiratory virus panel.

**Table 3:** Incidence of viral ARI and pneumonia per 100 child-years in a cohort of children under 2 years of age in a low-income urban community, Dhaka, Bangladesh (May 2015–February 2018)

Viral Pathogens	ARI per 100 Child-Year (95% CI)				Pneumonia per 100 Child-Year (95% CI)			
	All Ages	0 to <6 Months	6 to <12 Months	12 to 24 Months	All Ages	0 to <6 Months	6 to <12 Months	12 to 24 Months
Overall	212 (202–223)	344 (310–383)	229 (208–251)	180 (168–191)	30 (27–35)	76 (60–95)	41 (33–52)	17 (14–21)
Respiratory syncytial virus (RSV)	22 (19–26)	24 (16–36)	26 (20–35)	20 (16–24)	5 (4–7)	15 (9–25)	8 (5–13)	2 (1–4)
Rhinoviruses (RV)	137 (122–154)	371 (244–564)	354 (287–454)	100 (87–115)	12 (9–16)	92 (42–206)	24 (13–43)	8 (6–12)
Human parainfluenza viruses (HPIV)	17 (15–21)	35 (25–49)	25 (19–34)	11 (8–14)	5 (4–7)	13 (8–23)	11 (7–17)	2 (1–3)
Influenza viruses	15 (12–17)	26 (18–38)	17 (12–24)	11 (9–15)	1 (0.6–2)	4 (2–11)	1 (0.5–4)	1 (0.3–2)
Human metapneumovirus (hMPV)	8 (6–10)	17 (11–28)	1 (0.3–4)	8 (6–11)	2 (1–3)	7 (3–15)	1 (0.7–4)	2 (1–3)
Adenoviruses	9 (7–12)	7 (3–15)	13 (9–20)	8 (5–12)	1 (0.5–2)	1 (0.5–7)	2 (0.5–5)	1 (0.5–2)
Multiple viruses	28 (25–32)	52 (39–68)	29 (22–38)	23 (19–28)	4 (3–6)	9 (5–18)	5 (2–9)	3 (2–5)

ARI = acute respiratory infection.

### Seasonal trends in respiratory virus circulation.

**Figure 3.**
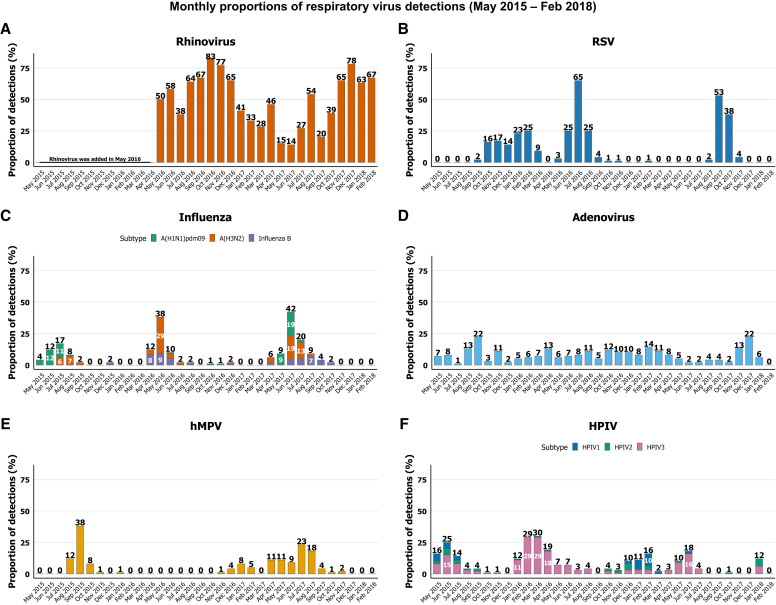
Monthly detection of viruses in children under 2 years of age with ARI or pneumonia (May 2015–February 2018).

The monthly distribution of respiratory virus detections showed clear seasonality. Viral activity peaked during the cooler months (November–February), with a secondary rise observed during the monsoon season (July–October). Influenza viruses and HPIVs showed peaks primarily during the monsoon season, whereas other respiratory viruses were more concentrated in the winter months (Figure 3).

## DISCUSSION

This prospective birth cohort study in a low-income urban community of Dhaka found a substantial burden of ARI and pneumonia among children under 2 years of age, with rhinovirus as the predominant viral pathogen, followed by RSV. In our cohort of children, the highest incidence of ARI and pneumonia was observed among those less than 6 months of age. Our findings further showed that respiratory viruses played a major role in childhood ARI and pneumonia, with viruses detected in ∼72% of all respiratory episodes. These results are consistent with prior studies conducted in diverse settings, including China, Algeria, and Bangladesh that have identified respiratory pathogens as primary contributors to ARI and pneumonia.^29^^–^^34^ However, most of these studies were limited to hospitalized children, thereby limiting their generalizability to community-based populations. Moreover, earlier community-based investigations frequently lacked comprehensive testing for the broad panel of respiratory viruses implicated in these conditions.^25^^,^^35^^–^^38^ By incorporating a broad panel of respiratory viral pathogens, our present study offers a more inclusive evaluation of their role in the burden and etiology of ARI and pneumonia in early childhood. These findings provide critical evidence to inform the development of targeted interventions aimed at reducing the incidence of these illnesses among children under 2 years of age.

RV emerged as the most frequently detected pathogen in the samples obtained from our cohort of children and was identified in over half of all ARI and pneumonia episodes. Although this high detection rate suggests a potential epidemiological significance, the clinical interpretation of RV presence warrants caution. RV is commonly regarded as a ubiquitous virus that can be detected in both symptomatic and asymptomatic individuals, and it is known for its persistence in nasal mucosal secretion for extended periods following infection.^33^^,^^39^ However, RV is not considered a colonizing virus because its persistence reflects extended viral shedding following acute infection. This prolonged shedding complicates the ability to attribute a direct causal role to RV in all instances of respiratory illness. Moreover, numerous community-based and longitudinal studies have documented RV detection in the absence of clinical symptoms, with rates ranging from approximately 11–64% in asymptomatic infants and young children.^40^^–^^42^ This absence of clinical symptoms further emphasizes the difficulty of attributing pathogenicity to mere RV presence. In our cohort, RV was more frequently co-detected in cases involving multiple viral pathogens. This pattern further complicates the interpretation of RV’s role in disease causation and raises the possibility that RV may be an incidental finding in some cases. Nonetheless, its substantial presence suggests that RV may still contribute to childhood respiratory illness either as a primary pathogen or through interactions with coinfecting viruses or host immune pathways. RV may also contribute to illness severity by acting synergistically with other respiratory viruses or by modulating host immune responses.^43^ Further investigations incorporating viral load measurements and host immune response profiling could help clarify the pathogenic potential of RV in early childhood ARI and pneumonia.

RSV was the second most frequently detected virus in our cohort, identified in 10% of all ARI episodes and in 17% of pneumonia cases. This finding aligns with a substantial body of global evidence recognizing RSV as a predominant cause of severe lower respiratory tract infections in young children.^44^^–^^48^ The disproportionate burden of RSV detected in pneumonia cases, particularly among infants under 6 months of age in our study underscores the critical need for effective preventive measures. Emerging strategies, such as maternal immunization during pregnancy and long-acting monoclonal antibody prophylaxis in early infancy, offer promising avenues for reducing RSV-related morbidity and mortality. There is an urgent need to consider the incorporation of these interventions into national immunization schedules in light of recent advancements in RSV vaccine development and novel immunoprophylactic agents.^49^^–^^52^ The need for such interventions is particularly important in LMICs such as Bangladesh, where the burden of RSV disease is substantial and access to viral diagnostics are limited. The implementation of such strategies could play a pivotal role in mitigating the impact of RSV on vulnerable pediatric populations.

Our analysis identified distinct seasonal peaks of both RSV and influenza virus detection among children under 2 years of age. We observed the highest activity for both viruses occurring during the monsoon and early post-monsoon months (May–September). This seasonal pattern aligns with previous studies from Bangladesh, which have frequently observed increased RSV circulation during the hot and rainy season, typically from mid-June to October.^53^^–^^56^ Furthermore, influenza activity has also been shown to peak between May and September, coinciding with high humidity and rainfall.^57^ However, both our findings and earlier research highlight considerable year-to-year variability in the timing and magnitude of RSV epidemics, with some years showing off-season activity, peaks during cooler months, or even biannual outbreaks.^54^ On the other hand, influenza seasonality appears to follow a more consistent pattern, as documented in both hospital- and community-based surveillance studies across the country.^58^ Both of these findings underscore the importance of continued viral surveillance and highlight the need to promote the timely administration of maternal RSV immunization and seasonal influenza vaccination before the peak season.

Our study has shown that the incidence of ARI has increased (212 episodes/100 child-years) in urban settings in comparison with previous estimates, which found the incidence of ARI to be 80/100 child-years among children under 2 years of age.^34^ Conversely, the incidence of viral pneumonia was somewhat lower in our study (30 episodes/100 child-years) in comparison with 40 episodes/100 child-years reported previously. Several factors, such as population density, environmental exposures (e.g., air pollution, indoor crowding), or seasonal patterns, may have contributed to the discrepancies between these two studies.^59^^–^^61^ Moreover, the lower pneumonia incidence might stem from variations in physicians’ judgment, cohort characteristics such as nutritional status, temporal changes in pathogen circulation, and health-care improvements between study periods. Despite these variations, both studies identified RSV and RV as predominant viral pathogens. This predominance further underscores the ongoing burden of viral respiratory illnesses in early childhood and emphasizes the need for targeted preventive strategies in similar low-resource urban settings.

Our finding of a higher incidence of pneumonia among children under 6 months of age aligns with observations from studies conducted in several African countries, China, and India.^32^^,^^62^^–^^64^ This heightened vulnerability in early infancy is likely attributable to a combination of factors, such as immature immune systems, limited physiological reserves, adverse socioeconomic conditions that may exacerbate exposure to environmental risk factors, and inadequate access to health care.^65^ Targeted public health interventions are essential to mitigate this risk. Evidence supports the promotion of prolonged exclusive breastfeeding, which provides optimal nutrition along with critical passive immunity through maternal antibodies and other immunological factors.^66^ In addition, maternal immunization during pregnancy, particularly against respiratory pathogens such as influenza and RSV has proven effective in reducing the burden of respiratory infections in young infants.^67^^–^^72^ This protection is primarily achieved through the enhancement of transplacental antibody transfer, which provides passive immunity during the early months of life.^73^ Implementing and expanding these strategies in resource-limited settings like Bangladesh could help protect infants during the early months of life when they are most vulnerable to severe respiratory illnesses such as pneumonia.

Our study has several limitations that should be considered when interpreting the results. First, the study population was drawn exclusively from an urban setting, which may limit the generalizability of our findings to rural populations. Second, we did not include radiological confirmation for pneumonia diagnoses, relying instead on clinical criteria based on the WHO definitions. Although this approach is practical and widely used in resource-limited settings, it may have led to misclassification or underestimation of pneumonia cases. Third, our diagnostic testing focused solely on viral pathogens, and we did not assess for bacterial etiologies of pneumonia, thereby limiting the comprehensiveness of our etiological attribution. Furthermore, the absence of a control group of asymptomatic children prevented us from distinguishing between asymptomatic viral shedding and true pathogen-related illness, which restricted our ability to draw causal inferences about the detected viruses. Moreover, although active follow-up was conducted through twice-weekly household visits, some samples may have been missed in instances where families were temporarily absent or primary caregivers were unavailable to accompany symptomatic children to the study clinic. Finally, we also couldn’t conduct risk factor analyses for ARI and pneumonia in our study, primarily because of the lack of a comparative group and the absence of pathogen detection data among healthy controls. Without these elements, it is difficult to robustly examine associations between host, environmental, or behavioral factors and disease outcomes. Future studies should incorporate well-defined control groups, include both viral and bacterial diagnostics, and consider imaging confirmation where feasible to better elucidate the multifactorial drivers of respiratory illness. Despite these limitations, our findings contribute valuable insights into the viral etiology and incidence of ARI and pneumonia in young children and underscore the ongoing burden of these conditions within a community-based setting.

The findings of this study highlight a substantial burden of laboratory-confirmed viral ARI and pneumonia among children under 2 years of age in a low-income urban community in Dhaka, Bangladesh. The observed incidence, particularly among infants under 6 months of age, underscores the vulnerability of this age group. RV and RSV were the most frequently detected pathogens, with RSV as a major contributor to severe disease. Despite the global availability of influenza and RSV vaccines, the absence of national vaccination policies targeting pregnant women and infants is a missed opportunity to reduce morbidity and mortality from childhood respiratory infections.

To address this burden, a comprehensive public health strategy tailored to resource-limited settings is essential. National immunization programs should prioritize the introduction of influenza and RSV vaccines, particularly for pregnant women, to confer passive immunity to infants during their most vulnerable months. Promotion of exclusive breastfeeding, maternal vaccination, and community education on early symptom recognition, hygiene, and timely health care–seeking behaviors are vital components. In addition, strengthening community-based surveillance and diagnostic capacity, along with research on coinfections and socioeconomic determinants, is also essential for effective prevention and control efforts.

## Supplemental Materials

10.4269/ajtmh.25-0466 Supplemental Materials
