# Supplementary material for: Incidence and Viral Etiology of Acute Respiratory Infections and Pneumonia among Children Under Two Years: A Birth Cohort Study in Dhaka, Bangladesh
Source: Am J Trop Med Hyg. 2025 Dec 4;114(2):237–46. doi: 10.4269/ajtmh.25-0466 (PMC12874757; doi:10.4269/ajtmh.25-0466)
Supplement: Supplemental Materials [file tpmd250466.SD1.pdf]

Table S1: Multiple viruses detected in ARI episodes (N=217) in children under-two years of age in a low-income urban community, Dhaka, Bangladesh (May 2015-February 2018)

**Combination of multiple viruses**	**Multiple Viruses detected in ARI episodes (N=217)**			
	**All ages n (%)**	**0 – <6 months n (%)**	**6 – <12 months n (%)**	**12 – 24 months n (%)**
Adenovirus+RV	87 (40.1)	19 (38.0)	13 (25.0)	55 (47.8)
hMPV+Adeno	4 (1.84)	3 (6.00)	–	1 (0.87)
hMPV+Adeno+RV	1 (0.46)	1 (2.00)	–	–
hMPV+RV	12 (5.53)	3 (6.00)	–	9 (7.83)
HPIV1+Adeno	4 (1.84)	1 (2.00)	–	3 (2.61)
HPIV1+Adeno+RV	1 (0.46)	–	–	1 (0.87)
HPIV1+hMPV	1 (0.46)	–	–	1 (0.87)
HPIV1+RV	2 (0.92)	1 (2.00)	–	1 (0.87)
HPIV2+Adeno	1 (0.46)	1 (2.00)	–	–
HPIV2+hMPV	1 (0.46)	–	–	1 (0.87)
HPIV2+hMPV+Adeno	1 (0.46)	1 (2.00)	–	–
HPIV2+RV	5 (2.30)	3 (6.00)	1 (1.92)	1 (0.87)
HPIV3+Adeno	3 (1.38)	2 (4.00)	1 (1.92)	–
HPIV3+Adeno+RV	3 (1.38)	3 (6.00)	–	–
HPIV3+hMPV	2 (0.92)	1 (2.00)	–	1 (0.87)
HPIV3+RV	22 (10.1)	3 (6.00)	7 (13.5)	12 (10.4)
RSV+Adeno	8 (3.69)	–	4 (7.69)	4 (3.48)
RSV+Adeno+RV	5 (2.30)	–	2 (3.85)	3 (2.61)
RSV+hMPV	1 (0.46)	1 (2.00)	–	–
RSV+HPIV3	3 (1.38)	1 (2.00)	2 (3.85)	–
RSV+HPIV3+RV	1 (0.46)	–	–	1 (0.87)
RSV+RV	49 (22.6)	6 (12.0)	22 (42.3)	21 (18.3)
**Total**	**217 (100)**	**50 (100)**	**52 (100)**	**115 (100%)**

Table S2: Multiple viruses detected in pneumonia episodes (N=30) in children under-two years of age in a low-income urban community, Dhaka, Bangladesh (May 2015-February 2018)

**Combination of multiple viruses**	**Multiple Viruses detected in Pneumonia episodes (N=30)**			
	**All ages n (%)**	**0 – <6 months n (%)**	**6 – <12 months n (%)**	**12 – 24 months n (%)**
Adenovirus+RV	7 (23.3)	–	2 (25.0)	5 (38.5)
Adenovirus+RV+Inf A/H	1 (3.3)	1 (11.1)	–	–
hMPV+Adeno	3 (10.0)	2 (22.2)	–	1 (7.69)
hMPV+RV	1 (3.33)	1 (11.1)	–	–
HPIV1+RV	1 (3.33)	–	–	1 (7.69)
HPIV2+Adeno	1 (3.33)	–	–	1 (7.69)
HPIV3+RV	6 (20.0)	2 (22.2)	4 (50.0)	–
RSV+Adeno	3 (10.0)	1 (11.1)	–	2 (15.4)
RSV+Adeno+RV	2 (6.67)	1 (11.1)	–	1 (7.69)
RSV+RV	5 (16.7)	1 (11.1)	2 (25.0)	2 (15.4)
**Total**	**30 (100)**	**9 (100)**	**8 (100)**	**13 (100)**
