# Supplementary material for: Incidence and Viral Etiology of Acute Respiratory Infections and Pneumonia among Children Under Two Years: A Birth Cohort Study in Dhaka, Bangladesh
Source: Am J Trop Med Hyg. 2025 Dec 4;114(2):237–46. doi: 10.4269/ajtmh.25-0466 (PMC12874757; doi:10.4269/ajtmh.25-0466)
Supplement: Supplemental Materials [file tpmd250466.SD2.pdf]

Title: Incidence and viral etiology of Acute Respiratory Infections and pneumonia among children under-two years: A birth cohort study in Dhaka, Bangladesh

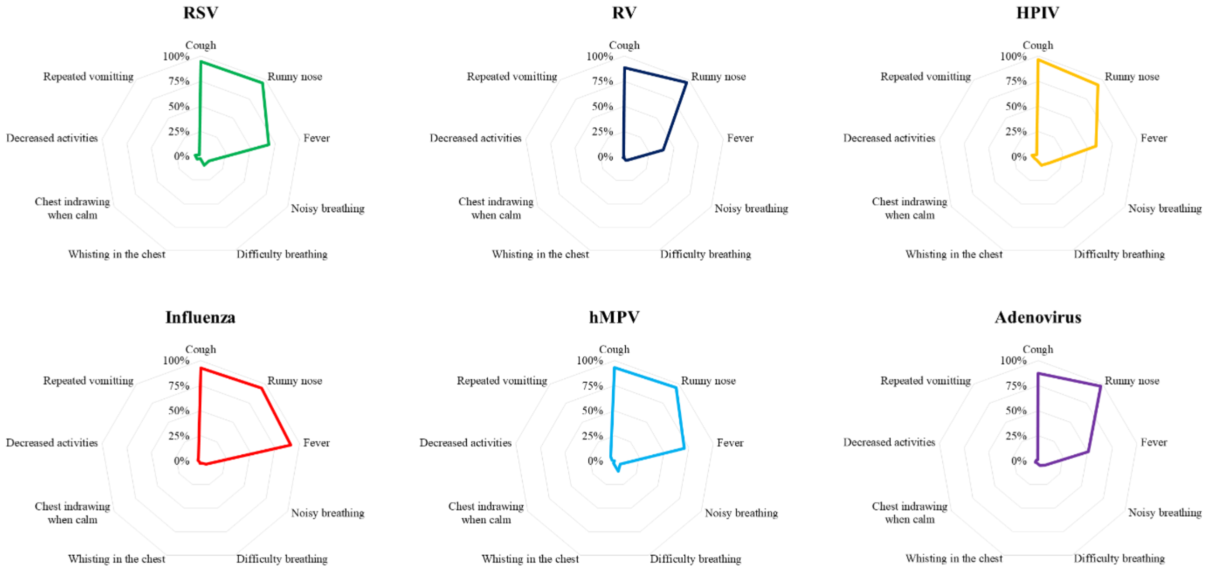

Supplemental figure 1: Symptom distribution by virus among children with viral ARI

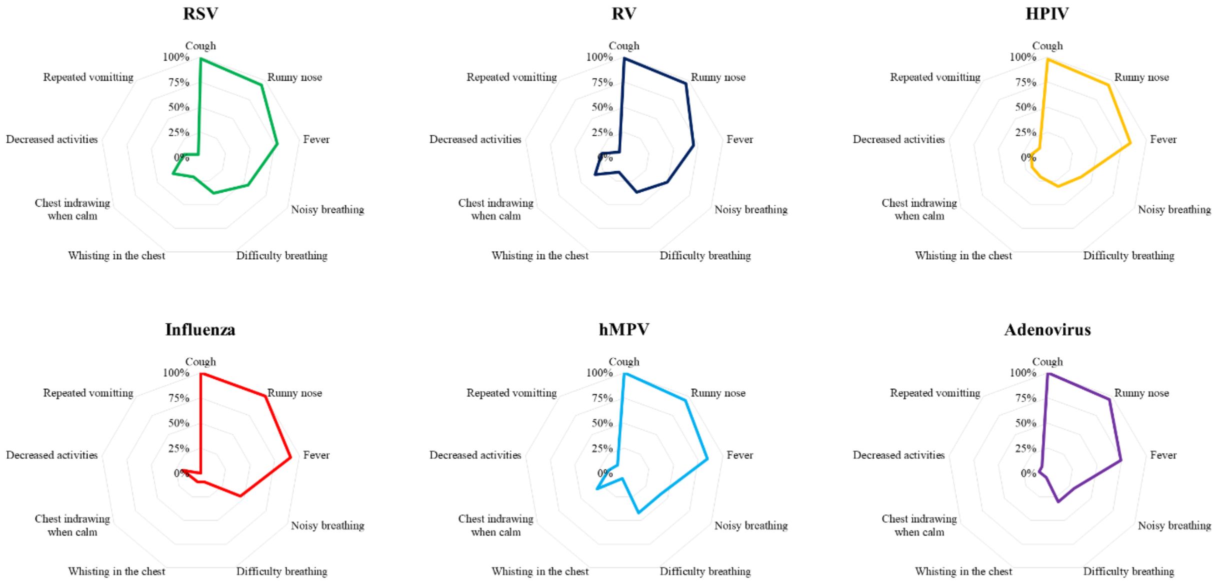

Supplemental figure 2: Symptom distribution by virus among children with viral pneumonia
